# Antibodies, repertoires and microdevices in antibody discovery and characterization

**DOI:** 10.1039/d3lc00887h

**Published:** 2024-01-02

**Authors:** Luca Johannes Schlotheuber, Ines Lüchtefeld, Klaus Eyer

**Affiliations:** a ETH Laboratory for Functional Immune Repertoire Analysis, Institute of Pharmaceutical Sciences, D-CHAB, ETH Zürich 8093 Zürich Switzerland klaus.eyer@pharma.ethz.ch; b ETH Laboratory for Tumor and Stem Cell Dynamics, Institute of Molecular Health Sciences, D-BIOL, ETH Zürich 8093 Zürich Switzerland

## Abstract

Therapeutic antibodies are paramount in treating a wide range of diseases, particularly in auto-immunity, inflammation and cancer, and novel antibody candidates recognizing a vast array of novel antigens are needed to expand the usefulness and applications of these powerful molecules. Microdevices play an essential role in this challenging endeavor at various stages since many general requirements of the overall process overlap nicely with the general advantages of microfluidics. Therefore, microfluidic devices are rapidly taking over various steps in the process of new candidate isolation, such as antibody characterization and discovery workflows. Such technologies can allow for vast improvements in time-lines and incorporate conservative antibody stability and characterization assays, but most prominently screenings and functional characterization within integrated workflows due to high throughput and standardized workflows. First, we aim to provide an overview of the challenges of developing new therapeutic candidates, their repertoires and requirements. Afterward, this review focuses on the discovery of antibodies using microfluidic systems, technological aspects of micro devices and small-scale antibody protein characterization and selection, as well as their integration and implementation into antibody discovery workflows. We close with future developments in microfluidic detection and antibody isolation principles and the field in general.

## Abstract 2

Essential in treating numerous diseases, novel therapeutic antibody candidates are needed. This critical review examines recent advances in microdevices and the challenges associated with candidate discovery and characterization.

## Graphical abstract

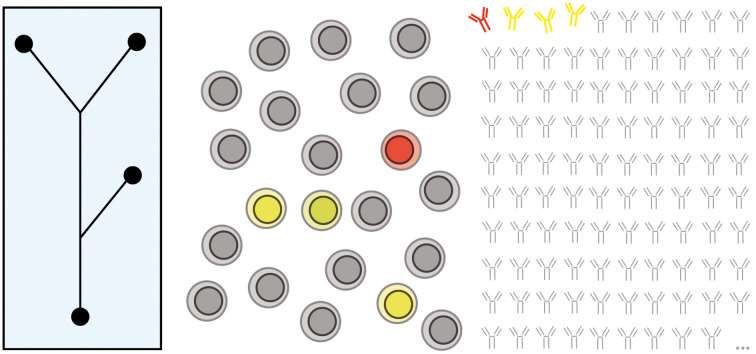

## Monoclonal antibodies as successful drugs and candidates

Monoclonal antibodies (mAbs) have emerged as a remarkably successful class of biologics extensively utilized across diverse disease categories, including oncology, infectious diseases, and auto-immunity.^1–4^ These molecules offer numerous advantages attributable to their exceptional selectivity, specificity, and binding affinity with a well-characterized and standardized development pipeline,^5^ complemented by their low toxicity owing to their resemblance to endogenous molecules.^6^ Notably, mAbs can further exhibit a range of potential secondary pro- and anti-inflammatory effector functions,^7^ encompassing neutralization of toxic actions, activation of the complement system and/or accessory effector cells, thereby offering a large range and resolution in the induced response. Indeed, the location of the antigen further dictates the range of potential secondary effects, such as complement deposition or cell-mediated killing, that require membrane-bound targets to be effective.^7^ The ability to manipulate the constant region of the antibody sequence allows customization of many of these functions,^8,9^ while the variable region, responsible for binding, induces various pharmacologically significant binding modes, spanning from simple binding to neutralization and from agonistic to antagonistic behavior, among others.^10,11^ The high selectivity and specificity of mAbs enable tailored recognition and differentiation between similar antigens or epitopes, even those containing single amino acid changes or specific post-translational modifications.^12,13^ These key attributes attract interest in the development of monoclonal antibodies to treat and diagnose a variety of complex diseases,^14^ and their versatility can be further increased by attaching different payloads.^15–18^ Given the potential sequence space for the antigenic binding side on the antibody, the identification and selection of a functional, developable and finally successful antibody sequence still remain challenging due to the large heterogeneity and the financial and clinical implications involved.

## Characteristics of successful antibodies

Due to their variability and various applications, defining the criteria for successful antibodies remains challenging. However, the success of an antibody therapy hinges upon its ability to correctly recognize the target antigen with optimal affinity and high specificity.^19^ With binding at its core, the antibody molecule should have access to the target antigen *in vivo*, induce the desired functionalities upon binding, be developable, producible, storable, allow for their formulation, and exhibit limited toxicity and side effects. To succeed as a drug, the medical benefit must justify its price, usually higher than small molecules. As of 2022, >246 antibody therapeutics were either approved, in regulatory review or undergoing late-stage clinical studies, according to Kaplon and co-authors.^20^ In oncology applications (120 antibodies), 97% of targeted antigens are membrane-bound, with the remaining 3% being secreted antigens. Conversely, 90% of the remaining antibodies target human proteins in non-cancer applications (126 antibodies), of which 67% are secreted and 23% are membrane-bound. Most remaining antibodies are designed to target infectious agents with the expectation of 2 antibodies targeting small-molecule drugs, binding molecules in case of an overdose.^20^

Various hurdles exist between antibody sequence identification and clinical (and commercial) success. While early-stage failures in antibody development are of various origins, considerable efforts have been made to predict antibody developability.^21^ These often focused on the antibody sequence. Expensive, catastrophic late-stage antibody failures have mostly been attributed to factors outside of the antibody sequence, such as errors in trial design, incomplete understanding of disease pathways and target roles, false biomarker identification, the generation of anti-antibody antibodies due to immunogenicity, suboptimal dosing and administration, as well as strategic and commercial considerations.^22^ Intriguingly, failure is only associated with the antibody sequence in the case of immunogenicity, where immune cells would be activated by specific sequences in the antibody structure. Almost all other factors are not directly connected with the antibody sequence, underlining the crucial role of clinical study design. Most important for the discussion here is that failures are often associated with the chosen antigen and the (miss)understanding of its role in the disease mechanism in antibody screenings. Additionally, the nature and location of the targeted antigen are important for every screening, as its presentation, confirmation, purity, and concentration will determine the outcome of the screen and the antibody that has been screened for. Exemplarily, many membrane proteins undergo extensive post-translational changes, for example, glycosylation or fragmentation due to shedding, complicating the selection of the target and the form it is present in the screening and *in vivo*. Choosing the correct antigen in the correct format and/or presentation remains crucial to antibody selections.

## Screening an antibody repertoire for candidates – single-cells, microfluidics, and repertoires

Therapeutic antibodies or candidates are often screened for in a diverse and heterogeneous repertoire of antibodies. Indeed, these repertoires, consisting of many individual monoclonal antibodies, are usually highly heterogeneous regarding their sequence and binding specificity to antigens. For example, the *in vivo* antibody heterogeneity stems from a complex process including activation, selection and differentiation of the cells towards present antigens, producing the effector subsets as antibody-secreting cells (ASCs), comprising plasma cells and plasmablasts, and memory cells (MCs) for long-term storage of the antibody information. Indeed, contact with an antigen produces many specific sequences that will be added in small fractions to the already historical repertoire.^23,24^ Therefore, diverse repertoires are available for antibody screening, each presenting its advantages and challenges, and Table 1 provides an overview of the most crucial characteristics of the commonly used antibody repertoires. Notably, a single-cell resolution is often required for these screenings, especially for natural B cell repertoires, aligning well with the general understanding that one antibody clone is produced by each individual B cell. Interestingly, the one-cell, one-antibody clone concept was first demonstrated by emulsifying cell suspensions in nanoliter droplets.^25^ Recent studies have challenged this observation and indicated that a smaller fraction of stimulated MCs may express more than one antibody,^26^ but the implications of this finding on antibody expression are not fully understood. In Table 1, we compared the different repertoires by the range of potential antigens, their *in* and *ex vivo* lifespan, the sample needed, whether the repertoire can be immortalized, and an estimated frequency of interesting antibodies in a sample, with estimated affinities. The last two points are highly variable, but the given numbers are calculated and summarized from a selection of papers and should only be seen as exemplary evidence in specific conditions.

**Table 1:** Common sources of antibody sequences in antibody screenings^156–170^

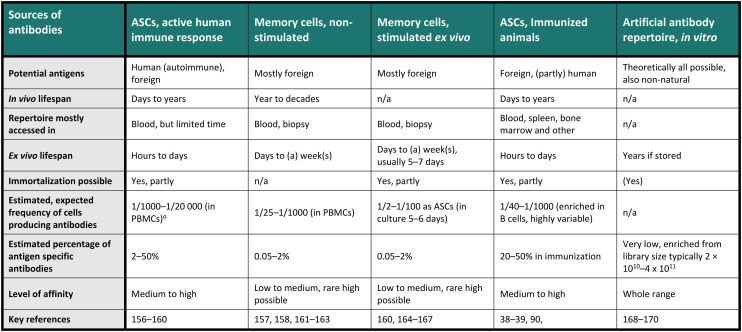

a Peripheral blood mononuclear cells (PBMCS, of which around 5–15% are B cells).

First, it is important to note that different subsets of antibody-producing cells can display different antibody repertoires.^27^ Due to the low frequency and short *ex vivo* lifespan of ASCs, for example, fast and high-throughput technologies are essential for efficient screening, although methods for the short-term culture exist.^28–30^ Solutions for long-term culture are also available, such as hybridoma or Epstein–Barr virus (EBV) immortalization, but only a small part of the B cells will successfully undergo transformation/fusion, and the collection of antibodies, respectively, will be present in these cells, potentially restricting diversity. Memory cells, on the other hand, display more consistent frequencies in human peripheral blood than ASCs, although their antibodies' median binding strengths to antigens are generally lower.^31–33^ However, a reasonably high throughput still allows extracting excellent binders from memory cells. Additionally, memory cells present the advantage of potential surface enrichment of antibodies and the ability to assess past infections in the donor, allowing sample collection independent of active infection.

Immunized animals constitute another prominent source for antibody screening, whether genetically modified or not. Various organs, such as the spleen, lymph nodes, bone marrow, and blood, can be accessed, each displaying differences in antibody repertoire present in ASCs and MCs.^34–36^ The quantity and quality of the induced repertoire in immunized animals highly depend on factors such as the chosen immunization protein (antigen), its quality, dose, administration, mouse strain, genetic model, harvest time, and immunization scheme, necessitating optimization for efficient and optimal screening^37,38^ (unpublished data), but the frequency of antigen specificity in total cells is usually higher than in humans. Nevertheless, appropriately chosen conditions can lead to identifying high-affinity antibodies, as the affinity distribution tends to follow a normal distribution with outliers in the high-affinity range.^39^ However, additional challenges are posed by ethical concerns, immunogenic constraints, and the different species that require antibody reformatting. Lastly, artificial antibody repertoires, *i.e.*, generated by randomization of sequences and often the basis for phage or eukaryotic display technologies, are utilized for screening, theoretically allowing access to all antigens (see Table 1). Although phage display has overcome certain challenges by screening libraries of antibody fragments against a potential antigen in multiple rounds, the technique has different limitations, such as false positive rates, low affinity and efficiency.^40^ Consequently, selecting the antibody repertoire to be screened for hits remains as critical as selecting the appropriate antigens for screening.

## Benefits and limitations of microfluidic technologies in the area of antibody discovery

The discovery of high-affinity antibodies is a crucial part of the drug discovery workflow, and although a vast diversity of tools, technologies and analytical pipelines have been developed, to this day, major challenges limit the detection of antibodies displaying high-affinity, -specificity or specific functionalities.^41^ Here, the rate of false positives and false negatives must be carefully matched to the required throughput and variability of the antibody sequences, and the integrated assays should screen for the desired functionality to name only two challenges. Especially for ASCs, a link between the secreted antibody and the cell has to be established, linking the information about its sequence with the displayed phenotype. This becomes even more complicated when complex functionalities are assayed on target cells, such as agonisms, antagonism or partial behaviors thereof. Nevertheless, antibodies can be sequenced from serum using mass spectrometry, but this is usually coupled with higher technical demands.^42^ These requirements are less important for MCs that can also be screened using standard cell sorters.^43,44^ In these cells, the antibody is presented on the cellular surface, and both the antibody and its sequence can be directly analyzed using fluorescence-activated cell sorting (FACS). However, this choice also limits the breadth of assays and functionalities that can be assessed. While FACS remains an important instrumentation in immunological laboratories to sort and separate individual events, this technique is less suitable to analyze secreted proteins and the cells secreting them.^45^

Interestingly, several requirements of antibody screenings overlap with the general advantages of a microfluidic workflow. Reducing volumes allows for faster screening of delicate cells and, combined with wells and droplets, links the antibody secretion to the secreting cell containing the genetic information. A small volume allows for a faster accumulation of nanomolar concentrations, a range where binding can be assessed – allowing faster processing of secreting cells displaying a limited lifespan. However, better control through laminar flow allows for tackling biological heterogeneity and standardization, which is especially important if more complex antibody functionalities are screened for parallelization and multiplexing increase the throughput to the required dimensions, and integration and automatization allow for standardized screenings. Lastly, microfluidic devices are often made of materials with attractive properties (*e.g.*, gas permeable, transparent, elastic), compatible with readouts such as fluorescence microscopy, and can even incorporate microelectrodes for bio-electrical applications. In addition, on-chip fluid shunting can be computer-controlled to generate automated systems.^46,47^

General limitations of microfluidic systems often lie in the low flexibility of changing conditions, either adding or removing reagents, nutrients, or waste thus limiting the time of culture/incubation.^48,49^ Moreover, microdevices require specific development and manufacturing expertise, are not always commercially available and are often not simple to use in a more biological or clinical setting, limiting their current applications in these highly demanding environments.^50,51^ Lastly, the throughput of multiple samples and large cohorts is often limited, as high throughput is often only achieved within one sample and automation and parallelization in terms of sample numbers are currently lacking.^52^

## Microdevices for the identification of animals and/or patients with suitable repertoires to screen

As a first step in discovering new therapeutic antibody candidates, individuals or animals with suitable repertoires for screening need to be identified. The current gold standard for measuring antibody presence and concentration in biological samples such as blood, plasma, or saliva is an enzyme-linked immunoassay (ELISA). ELISA employs serial dilution of the antibody sample that is then bound to an antigen-functionalized surface and binding is subsequently quantified by a fluorescently labeled detection antibody, reaching detection limits of around 100 pg mL^−1^. While simple, this method is material- and time-consuming, requires several washing steps, does not provide an instant readout, or yields any information on functionalities or epitopes.^53^ To reduce equipment cost, manual labor and required sample volume, the ELISA assay has recently been automated and miniaturized using liquid handling devices and a functionalized microfluidic chip.^54–58^ Interestingly, classical ELISAs can be used for various antigens, either soluble or antigens presented on the cell surface, defining a flexible screening platform that is highly reproducible, standardized and can be adapted in throughput to the repertoire study in question. ELISA remains the most often applied screening tool in front of repertoire analysis.

Alternatively, to enable point-of-care testing without the need for bulky equipment, lateral flow assays (LFA) were developed, as recently also experienced through SARS-CoV-2 rapid tests for different applications. In LFAs, which could be considered a microdevice by itself, a sample is loaded onto the test strip, and the accumulation of gold-conjugated detection antibodies on the antigen stripe leads to a local change in color that can be discerned by eye in order to confirm the presence of the antibody (or if set up differently, antigen) in the sample.^59^ This method allows for fast and easy testing but does not quantify the concentration of the antibody due to low standardization. Furthermore, mostly only soluble antigens can be employed in this technique, and the setting-up of the technology requires considerably more effort than a simple ELISA. Several advancements have been made employing the principle of LFA in microdevices to improve the detection and quantification of antibodies in serum samples.^60^

**Fig. 1:**
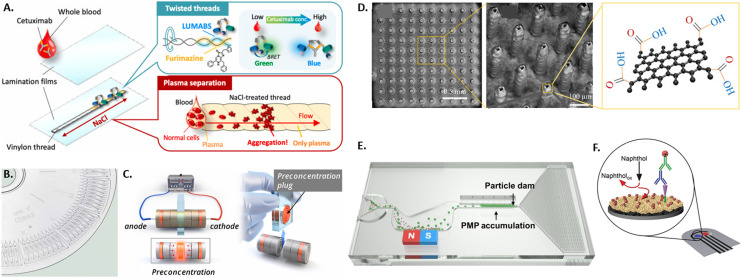
Overview of various microtechniques used to measure antibody presence in patient samples by A) a thread-based bioluminescence assay,^61^ B) a centrifugation disk for antibody detection,^64^ C) an electrokinetic disk for antibody pre-accumulation,^66^ D) graphene-coated 3D electrodes,^80^ E) microparticle accumulation at particle dam,^72^ and F) a gold nanoflower-based electrochemical sensor.^77^

Different microdevice technologies have been developed to accumulate the analyte at the testing site, enabling upconcentration and measurements of rare antibodies. Especially when antibodies are present at low frequencies in individuals, accumulation before screening represents a beneficial concept performed in microfluidic devices. Due to up-concentration, the screening displays increased sensitivity. Recently, proposed systems have used paper, threads, affinity membranes, centrifugation, and electrokinetics for the accumulation of the analyte. Unlike the standard paper-based lateral flow assays, thread-based assays were developed, where the liquid is transported to the testing side by capillary forces without additional equipment (see Fig. 1A).^61,62^ An advantage of using thread instead of paper is the versatile shaping of threads by twisting or sewing and the smaller sample volume due to the smaller surface area. Another approach for accumulating antibodies at the testing site is using functionalized membranes in vertical flow assays.^63^ For this, a porous alumina membrane was coated with polyelectrolyte layers and spotted with antibody-capturing mimotopes. The samples are then vertically passed through the membrane containing a microfluidic chip by gravity, the antibody is captured at the functionalized sites, labeled by a detection antibody, and quantified using fluorescence. Likewise, other approaches used in bulk protein accumulation, like centrifugation, can be utilized in microfluidics with bead-based bioreagents for antibody detection.^64^ Here, the analyte and assay reagents are added in the center of a disk containing various microfluidic channels and a chamber for mixing analyte and reagents. This technology has been commercialized and integrated into a compact disk (CD) format (see Fig. 1B). To avoid the need for specialized equipment to drive the rotation, a microdevice was developed with hand-powered rotation and centrifugation, further simplifying the process.^65^ Furthermore, electrokinetics can pre-concentrate the analyte before its quantification in a standard serological assay.^66^ For this, paper stripes were rolled up into disks and stacked into a cylindrical case for sample uptake. The outer paper disks on each side were previously coated with an ion-selective porous membrane and subsequently connected to an electric field (see Fig. 1C). The resulting differential electrokinetic movement of the sample components leads to a concentration of antibodies in the center of the paper disk stack.

The long assay times caused mainly by diffusion kinetics pose a disadvantage for bulk ELISA. Employing microfluidics offers inherent advantages due to lower dimensions and shorter diffusion times. To reduce the detection times, commonly limited by diffusion, a bead-based immunoassay was developed that allows for near real-time detection of antibodies.^67^ This was enabled by flowing functionalized beads, analyte and detection antibodies through serpentine-shaped microfluidic channels, thus creating chaotic advection and rapid mixing. Antibody presence can then be confirmed in near real-time by measuring the localized fluorescence intensity on the flowing beads. In order to enable the simultaneous and cost-efficient measurement of a large number of samples, *e.g.*, for the antibody serology of a large patient cohort, spotting assays were developed.^68^ Here, a microfluidic chip was developed consisting of 1024 individual measurement compartments, each divided into an immunoassay chamber and a sample chamber where the patient sample is spotted before the assembly of the fluidic layer of the chip. In the immunoassay chamber, surface functionalization and detection are performed by flowing different solutions through various microfluidic channels and valves.

In order to improve the ease-of-use and/or sensitivity of the readout of the presence and concentration of antibodies in patient blood or plasma, several new microtechnological approaches have been developed, integrating either optical or electrical readouts. The state-of-the-art readout of antibody presence in standard lateral flow assays is based on the aggregation of gold nanoparticles. Plasmonic enhancement of gold nanoparticles leads to the appearance of a strong red-colored signal. This allows readout by eye without the need for further equipment but does not enable quantification of antibody concentration in the sample and can yield ambiguous results at low antibody concentrations.^69,70^ One recent approach to better quantify antibody concentrations was developed by preloading and immobilizing the antibodies in the testing chamber of a microfluidic chip and subsequently adding fluorescent polystyrene particles.^71^ Using a smartphone-based optical readout, antibody concentration in saliva was quantified by either counting the number of unbound free-floating particles or measuring the capillary flow velocity affected by the number of unbound particles. Following a similar principle of measuring unbound particles, a magnetic microfluidic system was developed by Wu and colleagues, where polystyrene microparticles functionalized with detection antibody will only bind to antigen-coated magnetic microparticles if the specific antibody is present (see Fig. 1E).^72^ The bound particle aggregates are then magnetically removed from the flow, while unbound particles are accumulated at a particle dam into a visually detectable line. To further decrease the detection limit to 80 pg mL^−1^, a microfluidic system was integrated with a reflective fiber optic probe that acts both as a light source and for sensitively detecting changes in the reflected light intensities.^73^ The testing site was coated with antigen-coated gold nano spikes that exhibit a shift in the localized surface plasmon resonance (SPR) peak when the refractive index changes due to antigen–antibody binding. High-sensitivity and multiplexed optical detection of antibodies is also possible using graphene oxide quantum dots (GOQD), that are functionalized with the antigen and integrated in a parallel microchannel array for 60 plasma samples, reaching a detection limit of 0.3 pg mL^−1^.^74^ Here, the sample antibody is bound to the GOQDs and detected by detection antibody labeled with a fluorescent probe that exhibits fluorescence resonance energy transfer (FRET) with the graphene substrate. In order to detect antibodies optically without the use of nanoparticles, a thread-based bioluminescent system was developed.^61,62,75^ Here, two intertwisted threads were deposited separately on the one hand with luminescent antibody sensing proteins (LUMABS) and on the other with its bioluminescent substrate furimazine. If antibodies are present in a patient's blood sample, a bioluminescence color shift from green to blue occurs, which can be detected and quantified by a camera or smartphone. For non-optical detection of antibodies in blood samples, a microfluidic electrochemical approach was developed using an electrochemical immunoassay, where a nitrocellulose membrane is functionalized with the antigen.^76^ To this, antibodies present in the sample can bind, subsequently binding an enzyme-labeled detection antibody that then triggers the oxidation of the enzyme's substrate. The charge of the resulting product was then detected by chronoamperometry with a stencil-printed carbon electrode. A similar principle increased the surface area of the electrode with electrodeposited gold nanoflowers and measured by differential pulse voltammetry (see Fig. 1F).^77^ Another electrical approach was based on the principle of a Coulter counter.^78^ Here, magnetic beads functionalized with a capture antibody are incubated with a patient's saliva sample to bind specific antibodies. This leads to changes in the beads' electrical properties, which can be measured using a microfluidic impedance cytometer. The unique properties of graphene have been exploited to increase the sensitivity of impedance-based approaches down to a limit of detection of 0.6 pg mL^−1^.^79,80^ For this, pillar-shaped electrodes were coated with reduced-graphene-oxide that were subsequently functionalized with antigens (see Fig. 1D). Specific binding of the sample antibody to the graphene induces changes in the electrical circuit's impedance that can be detected by impedance spectroscopy.

## Overview of technologies enabling microfluidic discovery of novel antibodies

The discovery and screening for high-affinity and functional novel antibody sequences are crucial to the antibody drug discovery workflow. While the microdevice detection methods described in the previous section confirm the presence of specific antibodies in a repertoire, complete discovery approaches aim to isolate the immune cells encoding and producing such specific antibodies to identify the antibody sequence of the desired phenotype. This information is required to produce the antibody light and heavy chain at a large scale as a recombinant protein, further characterize the antibody, and develop the candidate into a therapeutic.

**Fig. 2:**
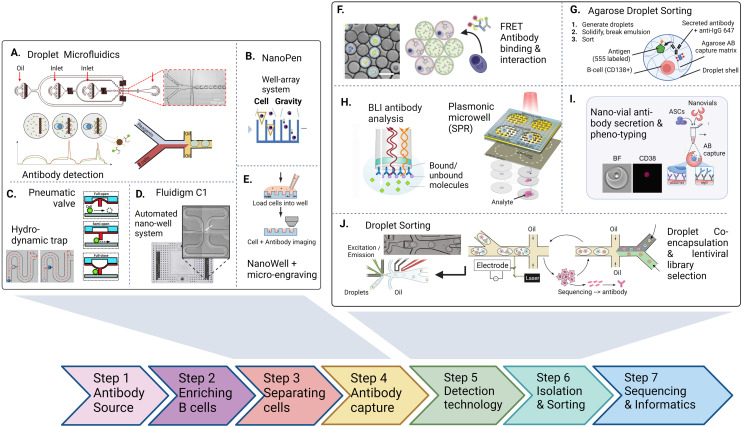
The antibody discovery process starting with the antibody source (either a biological, clinical sample or an artificial repertoire. After optional enrichment of cells, antibody or repertoire producing cell are first separated (A–E) inside water–oil droplets, trapped using pneumatic valves or settled in wells inside microfluidic chips to achieve single-cell resolution. 2nd, antibodies are captured, detected and characterized inside the trapped vessel (F–I). After characterizing (binding, interaction, activation, affinity, function), non-integrated technologies retrieve cells and antibodies for pooling and sequencing. Integrated approaches aim at direct detection and sorting of antibody producing cells in a continuous fluidic system (J). Figures adapted from A),^90,155^ B),^109^ C),^125,174^ D),^47^ E),^107^ F),^91^ H),^115,116^ I),^121^ J).^100,175^

Most general antibody discovery platforms usually start with approaches that separate antibody-producing moieties into individual containers to diverge the spectrum of present antibodies into their monoclonal form.^81,82^ After isolation and antibody detection, the next key step in the discovery is the isolation, sorting or physical removal of cells producing antibodies of interest from the analyzed repertoire (see Fig. 2),^83^ and certain techniques, such as antibody display, directly start at this step. Lastly, common to all techniques, the antibody sequence has to be determined using next-generation sequencing of transcripts encoding the antibody. Throughout these steps, the advantages of microfluidic technologies, as mentioned above, nicely fit the technical requirements, and therefore, many advances in the field of antibody discovery have been performed in microfluidic environments. Here, pico-sized reactors allow for fast detection of antibodies, compartmentalization on the single-cell level, full or partial automatization and combination to high-throughput detection, all benefiting the challenging and complex endeavor of finding novel candidates. Microtechnologies for antibody discovery can be differentiated into categories based on their separation technique: droplets, wells and valves, a categorization we will use in the following. Overall, within each separation category, a remarkable diversity of applications, incorporated detection principles, automation principles, data analysis workflows, fluidic designs, and downstream isolation protocols have been developed in recent years. Each separation technique exhibits its dis- and advantages, summarized in Table 2.

**Table 2:** Overview of key microfluidic antibody discovery technologies. Columns signify features of different discovery workflows: rows display platform technologies divided into droplet, nano-vial, well- and valve-based technologies. Additionally, key signature features are highlighted in orange (low performance) and green (high performance). Significant drawbacks spanning all technologies within a subsection are described on the far-right column. nM: nanomolar, refers to antibody concentrations which can be detected using the technology (range)^171–173^

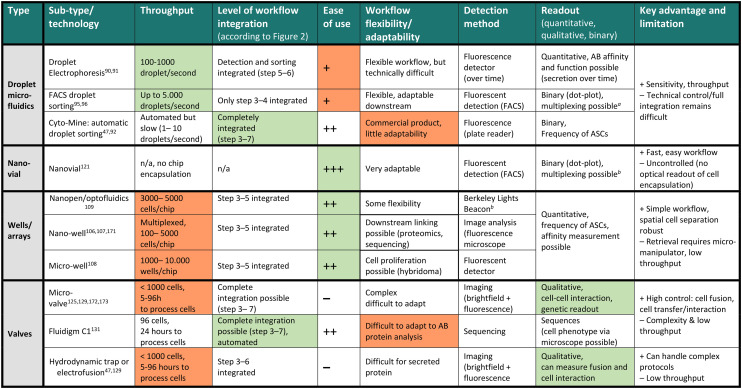

a Refers to adding phenotype or additional markers to discovery workflow when using cytometric sorting as a cell isolation method.

b Berkeley Lights Beacon is a commercially available, integrated cell-culture and imaging platform. AB: antibody, FRET: detection method *via* Förster resonance energy transfer, FACS: detection and sorting method: fluorescent activated cell sorting.

### Droplet microfluidics

Droplet microfluidics is a commonly used technique to separate the heterogeneous population of antibody-producing cells into individuals, where the cells are encapsulated into small volumes within an emulsion. Droplet microfluidics potentiates antibody discovery by allowing for the high-throughput generation of droplets containing cells and reagents. Within droplets, it is easy to combine sensitive readouts and allow for intracellular, membrane, and secreted protein analysis and quantification^84^ and the reagents for detecting antibodies are mostly antibody-based as well. However, upon antibody production, the signal of the droplet needs to be altered, making enzymatic probes less useful. Exemplarily, Mazutis *et al.* performed antibody capture in droplets using a single streptavidin-coated polystyrene bead modified to capture antibodies of interest.^85^ The antibodies of interest were secreted from single hybridoma cells in the droplets, and this setup allowed for measuring IgG secretion from all correctly encapsulated cells and was coupled to subsequent sorting. Signal change was measured as a fluorescence relocation of a secondary detection antibody onto the capture bead. Combining this workflow to detect antibody secretion with antigen-specificity further potentiates this technology as it allows making qualitative distinctions (specific/non-specific) upon the antibody secreted.^86^ Although with regard to sensitivity, droplets can be out-performed by bulk technologies which make use of signal amplification, down to femto-molar ranges, droplets are still detecting antibody in nano to sub-nanomolar concentrations which is suitable for protein secreted from a single cell.^87^ Shembekar *et al.* further demonstrated that antibodies targeting specific antigens on the cellular surface can be screened in this principle by co-encapsulating OKT9 hybridoma cells together with K562 leukemia cells expressing the transferrin receptor.^88^ While this approach does not enable the screening of all antibody-secreting cells as only antibodies binding to surface receptors are detected, the authors could show that using the cell itself as an antibody capture matrix enables the identification and enrichment of single hybridoma cells secreting transferrin-receptor specific antibodies at high throughput.

However, one downside of co-encapsulation of bead or cell together with the ASCs lies in the low co-encapsulation efficiency driven by the inherent principle of Poisson distribution of bead and cell. Eyer *et al.* and Gérard *et al.* overcame this issue by encapsulating more than 1000 magnetic nanobeads per droplet, assuring that each droplet will have similar bead numbers and active surfaces.^89,90^ These nanoparticles were modified to capture antibodies within each droplet, allowing detection in every droplet. This approach allowed for measuring IgG secretion and antigen specificity and affinity from each cell using a fluorescently labeled antigen in the assay (see Fig. 2A). The authors benchmarked their platform by detecting, sorting and single-cell sequencing of tetanus- and tetraspanin-8-specific antibodies with high efficiency, even though sorting speed with 600 events per second still remains the bottleneck of this study.

While most antibody assay technologies inside droplets rely on particles to capture the secreted antibody and measure fluorescence relocation for its detection, FRET represents another principle to detect antibodies of interest. FRET measures the energy transfer from an acceptor to a donor fluorophore when nearby, a principle used by Rutkauskaite and colleagues in their study.^91^ By using anti-c-myc peptide-specific antibodies, the authors have shown that a ternary complex is formed between the secreted antibody, the fluorescently-labeled antigen (FRET acceptor: c-myc-647) and the antibody detection probe (FRET donor: anti-IgG-488) which results in a FRET fluorescence signal. Without a specific antibody present, the two proteins are not in close proximity, resulting in no or little FRET. This assay further enabled the discrimination of membrane from secreted antibodies, could detect and quantify antibody secretion from individual hybridoma cells, and was also used for downstream sorting of positive antibody droplets. FRET has been established as a powerful detection principle that also can be applied in droplets, with sensitivities in the range of 5 to 50 nM.^92,93^

Whereas measuring inside droplets allows for high-throughput and sensitive detection, it also requires sorting to discriminate positive (antibody present) from negative droplets. Most of the named examples above have used such an approach. However, such sorting platforms need specialized equipment and personnel to run and are not readily available in most laboratories. Several developments have been made to approach this bottleneck to improve the ease of use and feasibility of droplet-based sorting without microfluidic sorters.^94^ Yanakieva *et al.* have made use of the thermoresponsive behavior of agarose.^95^ They have demonstrated that encapsulating cells and reagents together with liquid agarose at 37 °C, where their system was liquid, allowing for not only capturing proteins inside droplets but also offering simple downstream sorting using FACS after solidifying droplets and breaking emulsions at 4 °C, where the droplets were solid. This study demonstrated how agarose solidification of droplets can increase the ease of use of droplet isolation protocols, and how novel detection principles, such as reporter activation, can be integrated into droplet microfluidic workflows. Fischer *et al.* applied this principle to antibody isolation by generating an agarose-antibody capture matrix.^96^ By similarly generating single-cell emulsions of droplets containing reagents and agarose matrix, they were able to identify and sort antibodies directed against different segments of the SARS-Cov2 spike protein, which were captured in the agarose matrix and could be easily sorted upon solidification. Other applications for FACS-based droplet sorting (also called fluorescent-activated droplet sorting (FADS)) include the generation of double emulsion pico-reactors.^97–99^ In this workflow, cells are encapsulated twice to generate water–oil–water emulsions, which are more robust and can be used in aqueous systems such as FACS equipment. In contrast to sorting on chip, FADS allows for sorting-speeds common to cell-sorters with up to a few kHz.^95^ However, empty droplets further lower the throughput of interesting events to sort.

One challenge of droplet microfluidic workflows for antibody discovery remains in retrieving antibody sequences at single-cell resolution. While 10x Genomics has been paramount in generating single-cell sequencing data, it requires identified droplets or cells to be pooled in a single aqueous phase for encapsulation to be efficient (5–10 000 cells per run), thus binning single-cell phenotype information. Second, this number of cells might not be reached, depending on the selected phenotype and throughput. Third, if an emulsion is used, this needs to be broken, and the cells must be purified before the second encapsulation for sequencing. De Jonghe and colleagues integrated pico-injection into the workflow to address this crucial issue and integrated droplet antibody detection/characterization with sequencing.^100^ In this system, positive droplets are sorted, and sequencing reagents are added into positive droplets using a micro-sized automatic syringe, dramatically increasing the downstream antibody sequence recovery, although requiring higher sort/lower throughput to be efficient. Cyto-mine, a commercially available, automated droplet sorting system was developed, which couples droplet generation to sorting into a micro-titer plate suitable for genetic screening.^92,93^ This approach enabled integrated generation and sorting speed of 300 droplets per second and was benchmarked to isolate single-cell secreting antibodies using a FRET-based bio-assay (see Table 2). Another commercially available system, developed by Atrandi Biosciences, was benchmarked for detecting virus-neutralizing antibodies by using in-droplet cell expansion (Hybridoma) and virus-reporter cell pico-injection to identify antibodies of interest.^101^ This platform not only demonstrated the discrimination of a new functionality (antibody neutralization), but also used a sensitive detection method (FRET) while also improving throughput (reported up to 10^4^ droplets per second). Overall, the platform promises high droplet manipulation by merging, splitting or electrophoretic sorting and multiplexing capabilities of droplet assays. Finally, HiFiBio has significantly accelerated the droplet antibody discovery field, for instance, with their platform CelliGo, which aims at a sophisticated, comprehensive analysis of expression and binding of antibodies inside droplets but also further focuses on droplet barcoding and sequencing.^90^

Finally, droplet antibody discovery can also include detection principles such as mass spectrometry (MS). Here, antibodies are encapsulated after up-concentration and analyzed using MS, which, apart from identification and purification, also has the potential to study antibody modification such as glycosylation or free cysteines which is an important parameter for developing an antibody therapeutic. With the increasing sensitivity of mass-spectrometric analysis workflows, this can also allow protein sequencing by mass-spectrometry *de novo* (from the protein without the antibody sequence), a technology which has already been reported for the discovery of antigens.^102,103^

In summary, droplets are advantageous as they offer versatility in assay development. Owing to the small volume, antibodies can be detected without washing steps in a fast and reliable manner. Furthermore, droplets can be employed for various applications and detection principles by modifying the bio-assay included in the aqueous phase accordingly. Finally, they allow for single-cell resolution, making the sorted and isolated antibody monoclonal. This feature of preserving clonality is important because of the generally high antibody repertoire diversity.^104^ Nevertheless, the widespread use and identification of highly-potent antibody clones in a polyclonal repertoire using these technologies remains challenging. First, throughput (and false-positives) remains a limitation, particularly when antibody frequencies can be extremely low (1 in 1 million cells, for example). While imaging readouts allow for the potential identification of doublets, false-positives events, and other potential issues, they further reduce the throughput. Also, small numbers of sorted events present a further bottleneck in the downstream analysis due to the low number of cells and often require complicated operations (emulsion break, for example) that might lead to cell loss. Additionally, antibodies are often sorted as a binary outcome (binding *versus* non-binding, according to a threshold), not taking into account the complexity of different functionalities and binding modes present. Lastly, the throughput of the current approaches remains another bottleneck as instruments often include droplet sorting, which presents fairly low sorting speeds ranging between 100 and 1000 droplets per second, directly linked to the limited number of cells that can be sorted and analyzed. Therefore, few technologies enable comprehensive characterization while maintaining droplet control and cellular barcoding altogether in one workflow, and even if they do, they usually require multiple instruments/encapsulations. Finally, there is certainly still a gap for automated data analysis, which links big data of single-cell antibody features to a droplet ID and its subsequent sequence or protein downstream (see Fig. 2 steps 1–7). Although it is possible to measure a large amount of features inside droplets, an ideal technology would enable multi-parameter measurement in a non-binary manner at real-time, allowing for automated selection and transcript recovery simultaneously at high throughput during all workflow stages.

### Well-based approaches

Micro-well, nano-well and array-based workflows are currently among the most common microtechnological screening platforms for discovering antibodies (see Fig. 2C). Well-based approaches are comprehensive as they allow for multiple markers and secreted proteins to be characterized in parallel.^105^ Among different sub-technologies, micro-well chips for antibody discovery can be manufactured by engraving arrays of micro-fabricated wells which trap individual cells in nanoliter medium. Glass bearings or seals, coated with capture reagents allow for antibody analysis, such as secretion rate and quantification. Ogunniyi *et al.* utilized this principle by confining antibody-secreting cells into an engraved micro-well array consisting of a coated epoxy-functionalized glass slide, which can be sealed for a short incubation time (<60 min).^81^ Upon removal, wells can be analyzed like an antibody-protein micro-array, allowing for multiplexed detection of antibodies and specificities as dots using a fluorescence detection system or microscope and thus allowing to link a position to a specific well ID. While this approach is not necessarily single-cell, it allows for revitalization and expansion protocols suitable for hybridoma antibody discovery workflows (see Fig. 2C).

Further technological advancements also allow the detection of antibodies at single-cell resolution using nano-wells and micro-engraved arrays, whereas antibody secretion, surface binding, and transport are measured.^106^ This workflow principle was also utilized by Esfandiary *et al.*, which used a mixture of CD19+/Calcein nano-well and micro-array staining protocols to detect antibodies against the two antigens SSA/R060 and SSB/LA, which are specific biomarkers for rheumatic diseases and screened patient-derived antibody-secreting cells (isolated from human PBMCs).^107^ This approach was also applied together with a self-sorting (not using cell manipulation by valves or optical triggers) microwell chip by Abali *et al.* for detecting, quantifying and studying the frequency of epithelial cell adhesion molecule-specific antibodies secreted from a hybridoma cell line.^108^ In this workflow, the authors achieved single-cell resolution by creating pores inside a 6400-well chip, which block the entry of any other cell and also allow for secreted protein to be perfused and analyzed in an ELISA principle in the array below (see Table 2).

Increasingly, well-based approaches aim to detect single cells with multiple functionalities and isolate potential hits downstream. Winters *et al.* recently demonstrated this principle using nano-pens, a microfluidic chip containing wells of one nanoliter size and well-embedded beads for antibody detection *via* an ELISA-type reaction.^109^ By coupling this chip to a Berkeley Lights Beacon, a commercially available instrument, nanopens can be analyzed in real-time, and positive cells can be re-positioned using optoelectrical methods. This technique uses photoconductors that gently repel the cell inside and outside of wells^110^ and can thus allow for subsequent sequencing of the ASC. Similar to nano-pens, Lu *et al.* developed a sub-nanoliter microchamber array that focuses on the parallel detection of antibodies or cytokines with an even lower (femtomolar) sensitivity and detection of up to 14 analytes in parallel, which also demonstrates the potential for investigating multi-protein secretion from a single cell.^111^ Another advancement in the realm of well-based techniques is the development of micro-capillaries, such as uSCALE, a honey-comb shaped array where cells can be loaded together with protein libraries to identify binders at 10 000 capillaries per second detection and are equipped with an extraction laser to retrieve cells for downstream analysis.^112^ The authors demonstrated the versatility of this array by measuring cells expressing fluorescent proteins, antigen-library binding, and enzymatic activity.^113^

Finally, even though micro-wells are limited in size and hence throughput, adaptions with automated medium exchange allow an increase in throughput up to 70 000 wells.^114^ Microwells have also been further adapted to employ novel detection techniques for interactions between antibodies and antigens, such as biolayer interferometry, a label-free technique to study antibody binding and affinity.^115^ In this specific application, the interference pattern of the prostate-specific antigen (PSA) was measured against an anti-PSA antibody inside a micro-well plate where the antibody–antigen interaction causes a measurable shift in the reflected light pattern depending on the number of molecules present on the biosensor surface. More prominently and recently, the ability to perform spatio-temporal analysis of cell secretion and antibody–antigen binding using plasma-resonance was adapted and developed for micro-wells analysis.^116,117^ In this approach, three-dimensional micro-well arrays were developed using lithographic printing of wells containing gold substrate and nanometric holes conjugated to receptors for specific analytes. Detecting differences in light polarization in proximity to single antibody-secreting cells allows for a highly sensitive (see Fig. 2H). While nano-pen and capillary-based systems have reached sensitivities in the picomolar concentration range,^109^ well-arrays that enable more complex antibody characterization still require higher concentrations of antibody for optimal use, such as 0.5–1 μg ml^−1^ (>1 nM) for SPR and 2.5 μg ml^−1^ for BLI analysis.^115,118^

Overall, micro- and nano-well approaches have become popular, particularly because of their ease of use, sensitivity and simplicity in design as well as the ability to multiplex measurements. While micro-manipulation in arrays remains a limitation of well-based fluidic designs, recent advancements in cell manipulation using optical triggers and approaches for automation and single-cell sequencing may further improve this field. Altogether, the physical array separation of cells presents both advantages and disadvantages. It is favorable over droplets because it allows each well to be easily linked to an ID for integrating transcript and antibody phenotype (function) data. On the other hand, the physical well also limits the throughput and manipulation. This disadvantage, which also limits the combination of systems to automated, liquid handling of wells, can present a bottleneck, particularly for investigating diseases where the frequency of target antibodies is extremely low or large sample sizes.^114,119,120^

On another note, recently, Cheng *et al.* proposed so-called nanovials to separate individual antibody-secreting cells, using crescent-shaped suspended particles as small wells for individual cells.^121^ Using these vials, the authors have combined the analysis of secreted proteins and the sorting of the individual-producing cells. Each vial is a micro-container with a specific cavity where the cells are settled and trapped while the secreted protein is caught in the surrounding matrix. Cell capture is done in solution by simple mixing and incubation, but requires adhesion of the cell.^122^ Within the vial matrix, bioreagents are immobilized, enabling the detection of secreted antibodies but also enabling the manipulation of the cell as the vial itself is permeable for antibody stainings such as CD138 and CD19, common immunological markers for antibody-producing cells.^121^ While this technique is very appealing for its simplicity and throughput, it may suffer from empty vials, duplets, or aggregates as the encapsulation or filling of vials is performed in bulk. Interestingly, the authors further showed that nanovials can be directly sorted by FACS equipment and sequenced using 10x Genomics, further integrating the antibody discovery workflow (see Fig. 2I). However, the experimental reports are still rare, and additional studies are needed to evaluate the robustness of this approach.

### Valve-based techniques

Finally, microvalve-based techniques are another major microdevice family used in antibody discovery.^123–125^ These systems allow for precise fluid control of cells and bioreagents within a complex microfluidic environment and have been used in very diverse applications, including proteomics, genomics, and for lab-on-a-chip applications to mimic physiological behaviors at small scale.

Microvalve-based systems consist of a microfluidic chip setup often constructed from polydimethylsiloxane (PDMS) containing switching valves for fluidic control. Usually, valve setups include control layers and pressure sensors and operate at the micro-meter scale. Valve setups can allow studying complex behaviors such as cell polarization by trapping cells in hydrodynamic traps together with barcoded nanobeads to capture selective secreted proteins while also enabling fluidic control, media exchange, and most importantly, cell isolation using pneumatically activated valves.^126^ Moreover, valve systems are ideal for downstream sorting and washing steps for antibody sequencing from hydrodynamically trapped cells because pneumatic valves are already implemented within the fluidic chip.^127^

Even though valve systems remain niche compared to droplets and wells in antibody discovery, several published applications demonstrate the power that precise fluidic control can exercise to detect and manipulate antibody-producing cells. Firstly, cell-paring and cell–cell interaction studies and cell–cell fusion, essential for generating hybridoma cells from antibody-secreting cells, can be done using valve-based systems (see Fig. 2D).^128^ Secondly, chromatin immunoprecipitation studies, usually only performed in bulk and at great time and reagents cost, were successfully performed in a valve fluidic setup at high throughput.^129^ This approach enables studying a detected antibody's ability to bind and precipitate a protein–DNA complex as an antigen, often called CHIP-grading functionality. Although this specific setup is unsuitable for antibody discovery, it highlights the technology's potential in handling complex multi-step protocols. Both valve and microwell techniques further excel in their ability to be multiplexed through repetitive washing and staining steps, allowing for a higher-dimensional analysis compared to droplets. Kartalov *et al.* described a valve workflow to investigate antibodies binding multiple antigens from the same sample.^130^ Moreover, valve systems pose an ideal solution for downstream sorting and washing steps for antibody sequencing from hydrodynamically trapped cells because pneumatic valves are already implemented within the fluidic chip.^127^ Such setups have already been commercially developed within the Fluidigm C1 system (see Table 2),^131^ allowing for downstream mRNA generation and antibody sequencing. Finally, Zhou *et al.* showed that combining droplet and valve technologies with soft-lithography and microchannel PDMS chips can allow for integrated fluidic enrichment of antibody-secreting cells from generated droplets.^132^

However, valve systems are also accompanied by drawbacks. Unlike droplets and microwells, they require a more complex valve fluidic chip, including in- and outlets for pumps and switches, and additional implementations for synchronized multi-channel fluidic control. Additionally, they do not provide high throughput and are not as easily scalable as well arrays or droplets. However, while retrieving cells from nano-arrays and droplets remains difficult, microdevices employing pneumatic valves will continue to be essential in developing fully automated, precise isolation of antibodies or antibody-secreting cells with a particular function.

## Microfluidic systems for the characterization of antibodies after selection

Due to the large heterogeneity and the throughput involved, there always remains a risk of false-positive selection of an antibody candidate. This fact, coupled with the potential financial implications, makes it imperative that selected clones are further characterized after sorting. In the last five years, only few microdevices have been developed in order to characterize the selected antibodies before putting effort into upscaling their production and testing. Approaches developed in earlier years have been reviewed previously also by Kopp and Arosio.^133^ The main antibody characteristics investigated by microdevices are binding kinetics and specificity, thermal stability, structure and charge variants, and optimal purification conditions.

**Fig. 3:**
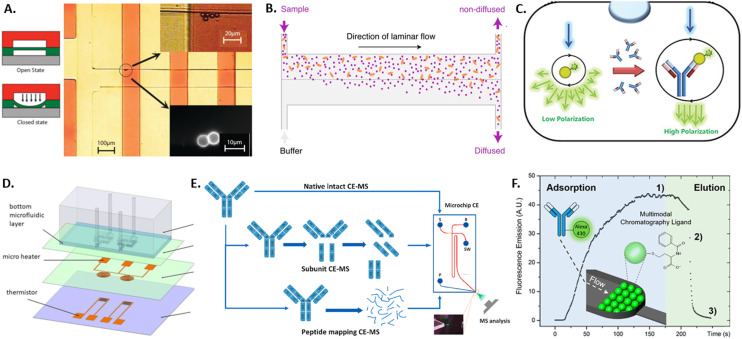
Overview of microtechnology approaches for antibody characterization by A) a sieve valve for particle trapping,^135^ B) a diffusion assay for antibody aggregates,^136^ C) a fluorescence polarization assay,^137^ D) MEMS-based differential scanning calorimeter,^140^ E) microfluidic capillary electrophoresis-mass spectrometry,^144^ and F) microfluidic multimodal chromatography.^149^

The main requirement of therapeutic antibody candidates remains in their specific binding to a target antigen. Therefore, studying the binding kinetics of an antibody candidate after selection is an important step in its characterization and can be performed by the standard techniques of ELISA (described above)^53^ or SPR. SPR detects small changes in the refractive index of a surface caused by the binding of the sample antibody with the use of surface plasmon waves that are triggered by an incident light source and captured by a photodetector.^134^ Due to the precise readout necessary, this technique requires large and expensive devices. Both techniques can quantify the binding kinetics only if the antibody concentration is known, require several 100 s of microliter of sample volume, and both suffer from surface effects such as non-specific binding and limited diffusion. Some of these limitations were overcome with recent microfluidic advancements, including sieve valves, lateral diffusion, and fluorescent polarization, as detailed in the following. By using a sieve valve as a reversible microfluidic trap for functionalized microbeads, the sample volume, detection limit, device requirements and costs can be greatly reduced compared to SPR (see Fig. 3A).^135^ Here, microbeads are functionalized with a capture antibody, loaded into microfluidic channels, and dynamically trapped using a sieve valve that allows the incubation and washing of the trapped particles. Thereby, the association and dissociation rates of the antibody–antigen complex can be measured fluorescently on single beads if the antibody concentration is known. In order to measure antibody affinity with unknown sample concentration microfluidic antibody affinity profiling can be employed (see Fig. 3B).^136^ Here, sample antibodies and fluorescently labeled antigens form a complex that is pushed into a microfluidic channel, while buffer solution enters from a parallel inlet, creating the parallel laminar flow of sample and buffer that allows for the size-dependent lateral diffusion of the sample complexes and the fluorescent determination of both the sample antibody concentration and its dissociation constant. Another concentration-independent approach for affinity determination is using fluorescence polarization (see Fig. 3C).^137^ Here, a sample antibody is co-encapsulated with fluorescently labeled antigen in water-in-oil emulsions. Upon illumination with polarized light, the polarization of the emitted light is measured, which corresponds to the size of the imaged complexes and thereby antibody–antigen binding. Since fluorescence polarization is intensity independent, this method does not depend on the antibody concentration, and does not require labelling of the sample antibody as in other approaches such as FRET. More microfluidic approaches analyzing protein binding have been reviewed by Arter *et al.*^138^

After the binding affinity and specificity of a therapeutic antibody is confirmed with the methods mentioned previously, further analysis is necessary to test antibody stability and charge variants. While standard techniques exist to measure each of these properties, many require large sample volumes and hence benefit from miniaturization by microtechnologies for pre-tests with low sample availability. Thermal stability of antibodies in solution is crucial to ensure conformational integrity, functionality and to prevent aggregation. It is classically measured using differential scanning calorimetry (DSC), which measures the transition temperature from the native tertiary structure to a denatured state by measuring the energy necessary to heat the sample to increasing temperatures and detecting transition temperatures.^139^ To reduce the measurement volume and time of DSC, a micro-electromechanical systems (MEMS) approach was developed consisting of two microfluidic chambers holding the sample and reference fluid, a microheater for scanning temperatures, and a thermistor for temperature measurement and feedback (see Fig. 3D).^140^

Degradation of antibodies can lead to loss of structure and functionality, which usually leads to changes in charge states within amino acid residues inside the antibody protein. To characterize such charge profiles and potential charge variants, these are classically separated and characterized by gel electrophoresis or chromatography. However, antibodies exhibit more complex charge heterogeneities than most proteins and, therefore, require complex separation and analysis that are complicated, time and material-intensive to perform with the classical methods. Microfluidic capillary electrophoresis mass spectrometry (CE-MS) overcomes some of these limitations by using a glass microchip with an integrated electrokinetic-based hydraulic pump that allows for electrokinetically-driven separation of a sample and electrospray ionization that directly couples to a mass spectrometer for mass and charge characterization (see Fig. 3E).^141^ Since the technology is commercially available,^142^ it has been used to characterize antibodies for critical quality attributes such as charge variants,^143,144^ peptide mapping,^145^ impurities and proteoforms.^146,147^ Similarly, the commercially available technique of microfluidic modulation spectroscopy allows for the characterization of the secondary structure of antibodies.^148^ Here, Fourier-transform infrared spectroscopy yields an absorption spectrum containing structural information. Microfluidic modulation enables the measurement of high-concentration samples and provides an internal reference, where the sample and a reference solution are flown into the spectroscopy chamber with continuous modulation of the flow ratio of sample to reference. In order to elucidate the 3D structure of an antibody, protein crystallization and subsequent X-ray diffraction are commonly applied. The optimization of the crystallization conditions for small nanoliter-sized sample volumes is enabled by a microfluidic chip composed of microchannels formed by water-permeable PDMS structures below an open reservoir. Here, the antibody and precipitant solution is filled into the microchannels and sealed as droplets by oil, while the top reservoir can be filled with salty aqueous solutions with varying water chemical activity. Permeation through the PDMS increases or decreases the droplet size and the eventual appearance and growth of crystals. Thereby, the optimal crystallization conditions for antibody solutions can be found in very small sample volumes. Once an antibody has been selected and characterized, there remain hurdles in manufacturing and purifying the antibody protein from cell culture supernatants, such as efficient antibody folding and chromatographic capture of the molecule of correct size and quality. This is normally performed by multimodal chromatography. Due to the possible interactions between multimodal ligands and antibody samples, large sample volumes would be necessary to test all variations using standard chromatography. Many conditions can be tested with low sample volumes using a microfluidic platform packed with chromatography bulk resins (see Fig. 3F).^149^ Additionally, the elution process can be monitored in real-time by fluorescence microscopy of the transparent microfluidic chamber.

Finally, the rheological properties of a final antibody solution need to be tested as they affect therapeutic safety, efficacy, and patient compliance. To test this crucial parameter before large batch production, a microfluidic diffusion chip allows for the rheological characterization with small sample volumes.^150^ Here, a small portion of high-concentration antibody solution is flown into a microfluidic chip filled surrounded by buffer or tracer particles. The distribution profile of the sample solution is then measured at subsequent positions in the microfluidic channel, giving insights on the rheology and viscosity of the sample solution.

## Future developments and outlook

In this review, our aim is to provide an overview of the challenges in the development of new therapeutic candidates, their repertoires and requirements. Therefore, we focused on antibody discovery using microfluidic systems, technological aspects of microdevices and small-scale antibody protein characterization and selection, and their integration and implementation into antibody discovery workflows. The field of antibody detection and discovery requires specialized technologies to capture the vast diversity of the antibody repertoire and to be able to measure if an antibody exerts a desired function. While, on the one hand, *in silico* modeling and artificial intelligence-based systems aim to model and predict antibody binding and will achieve these feats likely within the next years/decade, micro-technologies have emerged as key platforms for identifying high-quality antibody candidates. Various solutions exist today to identify high-affinity antibodies. As detailed above, some are still in the laboratory development stage, while others are fully commercialized.

For identifying a suitable repertoire containing the antibodies of interest, many different microtechnological approaches have been proposed for the pre-concentration of the sample and the sensitive and quantitative readout of antibody concentration. However, most assays do not allow for straightforward adaptation to different antigenic formats, and few allow inexpensive and uncomplicated use in a point-of-care setting.

Regarding the discovery and extraction of specific antibodies, apart from incorporating demanding feature characterization techniques into the detection process, increasing efforts are being made to automation, throughput and detection while enabling end-to-end processes that conserve the cell sequence information. Each principle feature for antibody discovery (droplets, vials, wells or valves) encompasses a different environment that allows for diverse applications rather than a one-size-fits-all situation. While droplet microdevices display high-throughput and are beneficial for discovery involving molecular barcoding or genetic screenings, fluidic manipulation (*e.g.*, immobilization or addition of bio-reagents), may pose challenges in the downstream isolation. On the other hand, nano- or microwell-based systems have limited throughput and scale, but offer simplicity, robustness and can offer powerful detection techniques such as plasma resonance. Finally, valve systems allow for handling complex protocols and offer precise fluidic control and infusion of assay reagents throughout the workflow. However, they have limitations in terms of scalability and throughput.^151^ Choosing a particular antibody discovery platform thus requires carefully considering the cost and benefits of each separation technique together with detecting a desired antibody function and its frequency in a biological repertoire.^47,82^ Overall, methods to increase sensitivity, specificity and/or multiple-parameter technologies are growing more dominant in microfluidics, for instance, by incorporating affinity or epitope mapping measurement into the screening process, as they allow a much more refined, qualitative search for potential candidates not only based on antigen binding alone.

In this context, even more technologies are being developed today that show great potential but have not yet been adapted to the field of antibody discovery. Such technologies include approaches aiming at microfluidic single-cell proteomics,^152^ microfluidic assays measuring virus neutralization, which could be utilized to detect antibodies affecting viral entry,^101^ as well as platforms attempting to measure the interaction between cells or receptors using microfluidic co-encapsulation.^153,154^ Finally, next-generation technologies that identify functionally active, agonist, antagonistic, or even bi-specific antibodies already exist.^116,155^ These workflows may enable high throughput cell–cell interaction studies to study the produced antibody and its communication with an antigen-expressing target cell, even in combination with CRISPR-Cas9.^153^ Apart from thus finding a much better class of antibodies for clinical development, this also means learning about the origination of the repertoire, which may answer much more profound biological questions as well.

For the characterization of selected antibodies, microtechnological approaches have mainly focused on quantifying binding affinity and stability. To enable a complete characterization at low volume scales, approaches addressing producibility, formulation optimization, and off-target binding will be valuable extensions. Furthermore, advanced microfluidic cell and organoid culture models might enable earlier and easier testing of the biological effect of the selected antibodies, including toxicity, off-target binding or anti-antibody formation.

Overall, microtechnologies pose an immense potential for the detection, discovery and characterization of therapeutic antibodies since many technological requirements of the workflow overlap with the advantages of microdevices. Here, many advances still lie ahead with the extension and combination of existing techniques, additional antibody functionalities, new antibody repertoires such as other formats or bispecific candidates, providing novel possibilities for new microdevices and single-cell approaches, and the incorporation of new computational and machine-learning-based analyses.

## Conflicts of interest

EK is the co-author of several patents in this field and the scientific co-founder of Saber Bio SAS, a French start-up company.

## Supplementary Material
